# The TP53 mutation rate differs in breast cancers that arise in women with high or low mammographic density

**DOI:** 10.1038/s41523-020-00176-7

**Published:** 2020-08-07

**Authors:** Dane Cheasley, Lisa Devereux, Siobhan Hughes, Carolyn Nickson, Pietro Procopio, Grant Lee, Na Li, Vicki Pridmore, Kenneth Elder, G. Bruce Mann, Tanjina Kader, Simone M. Rowley, Stephen B. Fox, David Byrne, Hugo Saunders, Kenji M. Fujihara, Belle Lim, Kylie L. Gorringe, Ian G. Campbell

**Affiliations:** 1 Cancer Genetics Laboratory, Peter MacCallum Cancer Centre, Melbourne, VIC Australia; 2 Sir Peter MacCallum Department of Oncology, University of Melbourne, Melbourne, VIC Australia; 3 Lifepool, Peter MacCallum Cancer Centre, Melbourne, VIC Australia; 4 Centre for Epidemiology and Biostatistics, Melbourne School of Population and Global Health, University of Melbourne, Melbourne, VIC Australia; 5 Cancer Research Division, Cancer Council NSW, Sydney, NSW Australia; 6 Sydney School of Public Health, University of Sydney, Sydney, NSW Australia; 7 BreastScreen Victoria, Carlton South, VIC Australia; 8 Department of Surgery, University of Melbourne, Melbourne, VIC Australia; 9 The Royal Melbourne and Royal Women’s Hospitals, Parkville, VIC Australia; 10 The Edinburgh Breast Unit, Western General Hospital, Edinburgh, UK; 11 Department of Pathology, Peter MacCallum Cancer Centre, and University of Melbourne, Melbourne, VIC Australia; 12 Drug Discovery Biology, Monash Institute of Pharmaceutical Sciences, Monash University, Parkville, VIC, Australia; 13 Cancer Genetics and Genomics Program, Peter MacCallum Cancer Centre, Melbourne, VIC Australia

**Keywords:** Cancer genomics, Breast cancer

## Abstract

Mammographic density (MD) influences breast cancer risk, but how this is mediated is unknown. Molecular differences between breast cancers arising in the context of the lowest and highest quintiles of mammographic density may identify the mechanism through which MD drives breast cancer development. Women diagnosed with invasive or in situ breast cancer where MD measurement was also available (*n* = 842) were identified from the Lifepool cohort of >54,000 women participating in population-based mammographic screening. This group included 142 carcinomas in the lowest quintile of MD and 119 carcinomas in the highest quintile. Clinico-pathological and family history information were recorded. Tumor DNA was collected where available (*n* = 56) and sequenced for breast cancer predisposition and driver gene mutations, including copy number alterations. Compared to carcinomas from low-MD breasts, those from high-MD breasts were significantly associated with a younger age at diagnosis and features associated with poor prognosis. Low- and high-MD carcinomas matched for grade, histological subtype, and hormone receptor status were compared for somatic genetic features. Low-MD carcinomas had a significantly increased frequency of *TP53* mutations, higher homologous recombination deficiency, higher fraction of the genome altered, and more copy number gains on chromosome 1q and losses on 17p. While high-MD carcinomas showed enrichment of tumor-infiltrating lymphocytes in the stroma. The data demonstrate that when tumors were matched for confounding clinico-pathological features, a proportion in the lowest quintile of MD appear biologically distinct, reflective of microenvironment differences between the lowest and highest quintiles of MD.

## Introduction

High mammographic density (MD) is associated with a significant increase in breast cancer risk^1,2^, with those in the highest quintile of MD having 4–6-fold increased risk compared to women in the lowest quintile of MD^3^. Within population-based breast mammographic screening programs, women with high MD experience an increased rate of interval breast cancer diagnosis^4,5^.

Few studies have explored the molecular landscape of cancers arising in low and high MD, with the majority of studies focusing on altered expression of a few molecules involved in extracellular matrix formation^6–10^ or single-nucleotide polymorphisms (SNPs) associated with both MD and breast cancer risk^11–13^. However, the additive effects of these common SNPs explain only a small percentage (<5%) of total MD variance with the remainder possibly attributed to unknown genes. Studies have investigated strong breast cancer susceptibility genes, *BRCA1* and *BRCA2*, and show no significant link with MD^14,15^.

While large repositories of genomic data are available for breast cancer, these data are seldom linked to MD scoring and consequently it is unknown if the molecular mechanisms of breast cancer development differ in dense versus non-dense breasts. An understanding of the underlying biological reasons why women with dense breasts are at a higher risk for developing breast cancer may identify opportunities to reduce that risk. The reason for the association between MD and breast cancer risk is currently unknown, although it is reasonable to presume that it reflects differences in the microenvironment. Consequently, if the drivers of breast cancer development in low- and high MD are different this might also result in breast cancers with divergent molecular profiles and provide evidence of a direct link between MD and breast cancer development. Therefore, the aim of this study was to determine the biological relationship of breast cancers arising in the lowest and highest quintiles of MD through somatic genomic analyses of a large cohort of well annotated breast cancers.

## Results

### Dense breasts exhibit a more aggressive phenotype

A total of 842 Lifepool participants were identified as having being diagnosed with a breast carcinoma (either invasive or in situ) where a mammogram preceding the diagnosis was available for calculation of an MD score. Among the 670 invasive breast cancers, 142 and 119 were categorized as arising in breasts of the lowest and highest MD quintiles, respectively (Table 1). Women diagnosed with an invasive breast cancer in the highest MD quintile had a significantly lower mean age at diagnosis (61.5 [range 43–81]) compared with the lower quintile (64.5 [range 47–88]) (*p* = 0.0007) (Table 1). Invasive breast cancers in the highest quintile were significantly more likely to be interval breast cancer diagnoses (*p* = 0.0006) and a self-reported strong family history of breast cancer (*p* = 0.0336) (Table 1). The intrinsic subtype distribution (according to St Gallen classification)^16^ (Table 1, Supplementary Table 1), invasive cancer histology subtype distribution, tumor grade, nodal involvement, and tumor proliferation (as measured by Ki67 staining) were not statistically different between cancers arising in the lowest versus highest MD quintiles (Table 1).

**Table 1:** Clinico-pathological features of breast cancers diagnosed in the lowest and highest quintiles of mammographic density.

Characteristics	Lowest quintile	Highest quintile	*P* value
Invasive	142	119	
Age at diagnosis			
Mean ± SD	64.5 ± 7.0	61.5 ± 7.5	0.0007^a^
Median	65.5	61.4	
Range	47–88	43–81	
Screening timing			
Lapsed screener	2 (1%)	1 (1%)	0.0006^b^
Interval cancer	10 (7%)	27 (23%)	
Screen detected	130 (92%)	91 (76%)	
Tumor size			
<20 mm	80 (60%)	57 (53%)	0.3966^b^
20–49 mm	39 (30%)	41 (38%)	
≥50 mm	13 (10%)	10 (9%)	
NA	10	11	
Intrinsic subtype			
TNBC	10 (7%)	6 (6%)	0.9450^c^
ER−, Her2+	2 (1%)	2 (2%)	
Luminal Her2+	8 (6%)	7 (7%)	
Luminal	114 (86%)	90 (87%)	
NA	8	14	
Invasive cancer histology subtype			
Ductal	117 (82%)	91 (76%)	0.3523^b^
Lobular	7 (5%)	11 (10%)	
Other invasive	18 (13%)	17 (14%)	
Tumor grade			
G1	34 (26%)	31 (29%)	0.4003^b^
G2	62 (47%)	55 (51%)	
G3	36 (27%)	21 (20%)	
NA	10	12	
Nodal status			
Positive	22 (21%)	19 (20%)	>0.9999^b^
Negative	82 (79%)	75 (80%)	
NA	38	25	
Proliferation index (Ki67)			
High (≥15%)	19 (25%)	23 (35%)	0.1976^b^
Low (<15%)	58 (75%)	42 (65%)	
NA	65	54	
First-degree relatives with breast cancer			
Yes	39 (27%)	46 (39%)	0.0637^b^
No	103 (73%)	73 (61%)	
Strong family history of breast cancer			
Yes	11 (8%)	20 (17%)	0.0336^b^
No	131 (92%)	99 (83%)	

Calculation of percentage within the lowest quintile and highest quintile cohort is presented within parentheses. *NA* data not available. ^a^Two‐tailed *t*‐test was applied. ^b^Two-tailed Fisher’s exact test. ^c^Chi‐square test was applied.

Among the 172 in situ breast cancers, 31 and 58 were categorized as arising in breasts of the lowest and highest MD quintiles, respectively. Age at diagnosis, screening timing, tumor size, grade, histology, estrogen receptor status, and family history were compared and showed no significant differences (Supplementary Table 2).

### Differences in somatic mutation profile

Targeted gene exon sequencing of 39 known breast cancer driver and hereditary predisposition genes, including 28 DNA repair genes (Supplementary Table 3), was performed on tumor DNA from all available lowest quintile (*n* = 29) and highest quintile breast cancers (*n* = 27), excluding in situ tumors (Supplementary Dataset). The cancers in each quintile were similar in age and grade (Supplementary Table 4).

Collectively, there were no significant differences in the somatic mutation frequency per Mb per tumor in the lowest MD compared with the highest MD (0.78 [range 0.21–1.91] versus 0.83 [range 0.11–4.55], *p* = 0.459), and this was not different when comparing within the luminal (*p* = 0.696), ductal (*p* = 0.403), and combined luminal/ductal (*p* = 0.461) subtypes (Supplementary Fig. 1). Luminal/ductal cancers were prioritized in the sub-analysis to remove the potential bias associated with lobular breast cancer diagnosis which can be masked in dense breasts^17^. The frequency of somatic mutations in each of the most commonly mutated breast cancer driver genes (*PIK3CA, MAP3K, KMT2C, TP53, GATA3, CDH1*, and *CBFB)* were similar between the two groups with the exception of *TP53* (Table 2). The frequency of somatic mutations in breast cancer driver genes between the lowest and highest quintile was also compared within triple-negative breast cancers and lobular breast cancers showing no significant differences but the number of cancers compared was low (Supplementary Table 5).

**Table 2:** Somatic driver mutation profile in breast cancers diagnosed in the lowest and lowest quintiles of mammographic density.

	Entire cohort (lowest *n* = 29, highest *n* = 27)							Luminal breast cancers (lowest *n* = 28, highest *n* = 24)							Luminal and ductal breast cancers (lowest *n* = 27, highest *n* = 15)						
	Lowest, *n* (%)			Highest, *n* (%)				Lowest, *n* (%)			Highest, *n* (%)				Lowest, *n* (%)			Highest, *n* (%)			
Gene	Lof	MS	%	Lof	MS	%	*P*	Lof	MS	%	Lof	MS	%	*P*	Lof	MS	%	Lof	MS	%	*P*
*AKT1*	—	2	7	—	—	0	0.4916	—	2	7	—	—	0	0.4932	—	2	7	—	—	0	0.5296
*ARID1A*	2	1	10	1	1	7	>0.9999	2	1	11	1	1	8	>0.9999	2	1	11	—	—	0	0.2944
*BAP1*	—	1	3	—	—	0	>0.9999	—	1	4	—	—	0	>0.9999	—	1	4	—	—	0	>0.9999
*BRCA1*	—	—	0	—	2	7	0.2279	—	—	0	—	2	8	0.2081	—	—	0	—	2	13	0.1220
*BRCA2*	1	1	7	—	—	0	0.4916	1	1	7	—	—	0	0.4932	—	1	4	—	—	0	>0.9999
*CBFB*	4	1	17	1	2	11	0.7066	4	1	18	1	2	13	0.7109	4	—	15	—	2	13	>0.9999
*CDH1*	2	1	10	5	—	19	0.4620	2	1	11	5	—	21	0.4466	1	1	7	—	—	0	0.5296
*CHEK2*	—	1	3	—	—	0	>0.9999	—	1	4	—	—	0	>0.9999	—	1	4	—	—	0	>0.9999
*GATA3*	2	—	7	2	1	11	0.6642	2	—	7	2	1	13	0.6521	2	—	7	1	1	13	0.6080
*KMT2C*	3	1	14	4	—	15	>0.9999	3	1	14	4	—	17	>0.9999	3	1	15	3	—	20	>0.9999
*MAP2K4*	1	—	3	—	—	0	>0.9999	1	—	4	—	—	0	>0.9999	1	—	4	—	—	0	>0.9999
*MAP3K1*	6	1	24	3	2	19	0.7482	6	1	25	3	2	21	0.7543	6	1	26	3	1	27	>0.9999
*MEN1*	—	—	0	—	1	4	0.4821	—	—	0	—	1	4	0.4615	—	—	0	—	1	7	0.3571
*NCOR1*	—	1	3	1	2	11	0.3434	—	1	4	1	2	13	0.3242	—	1	4	—	1	7	>0.9999
*NF1*	—	—	0	—	1	4	0.4821	—	—	0	—	1	4	0.4615	—	—	0	—	—	0	—
*PIK3CA*	—	16	55	—	8	30	0.0644	—	16	57	—	8	33	0.1025	—	15	56	—	5	33	0.2087
*PTEN*	1	1	7	1	—	4	>0.9999	1	1	7	1	—	4	>0.9999	1	1	7	1	—	7	>0.9999
*RUNX1*	—	1	3	1	—	4	>0.9999	—	1	4	1	—	4	>0.9999	—	1	4	1	—	7	>0.9999
*SF3B1*	—	1	3	—	—	0	>0.9999	—	1	4	—	—	0	>0.9999	—	1	4	—	—	0	>0.9999
*TBX3*	3	—	10	—	—	0	0.2373	3	—	11	—	—	0	0.2398	3	—	11	—	—	0	0.2944
*TP53*	3	6	31	1	1	7	**0.0420**	2	6	29	—	—	0	0.0051	2	5	26	—	—	0	0.0772

A two-tailed *p* value was calculated. Bold *p* values highlight somatic mutations that were significantly different between low- and high-MD breast cancers.

**Fig. 1:**
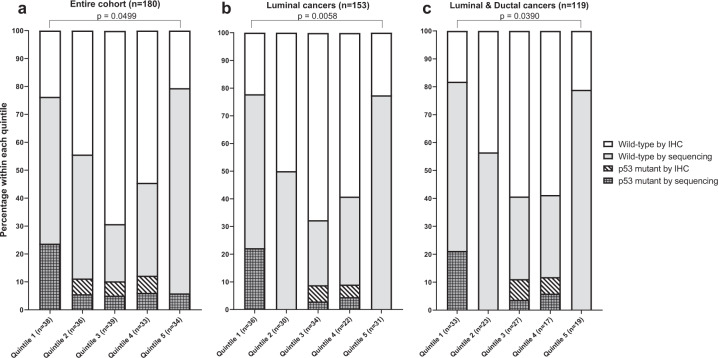
p53 mutation analysis in breast carcinomas arising in all quintiles of mammographic density. Barplots showing the percentage of cases within each quintile of MD that were either mutant or wild type for p53, scored for either **a** the entire breast cancer cohort, **b** luminal cancers only, and **c** combined luminal subtype and ductal histology cancers.

A limitation to the *TP53* mutation finding is that it is based on small numbers, and tumor sequencing was prioritized to samples within the highest and lowest quintiles of MD, and as such the *TP53* mutation frequency in the intermediate quintiles was unknown. To address this issue, immunohistochemistry (IHC) for p53 expression was performed on tissue microarrays (TMAs) prepared for 180 breast cancers that encompassed the entire quintile range of MD. Cases within each quintile of MD were determined to be either mutant (p53 absent, overexpression, or cytoplasmic staining in cancer cells) or wild type for p53. Of the cases analyzed by IHC, 80 overlapped with those cases that had targeted sequencing, and the concordance of *TP53* status with IHC was 79/80. The discordant case (Lifepool ID 13198, Supplementary Dataset) which is a quintile 5 metaplastic TNBC was due to the >80% stromal cells within the two independent tumor cores. Among the entire cohort (Fig. 1a), luminal (Fig. 1b) or luminal/ductal subtypes (Fig. 1c), p53 mutations were observed at a significantly higher frequency in breast cancers from the lower quintile compared to the highest quintile MD group (24% versus 6%, *p* = 0.0499; 22% versus 0%, *p* = 0.0058; and 21% versus 0%, *p* = 0.0390, respectively).

Clinico-pathological and genetic features were compared between *TP53* mutant versus *TP53* wild-type cases in the lowest quintile of MD. *TP53* mutant cases were significantly larger (*p* = 0.0354) and were of higher grade (*p* = 0.0028) compared to *TP53* wild-type cancers (Supplementary Table 6). No significant differences in somatic driver gene mutations or DNA repair gene mutations (excluding *TP53*) were observed (Supplementary Table 7).

### Somatic copy number aberration differences

**Fig. 2:**
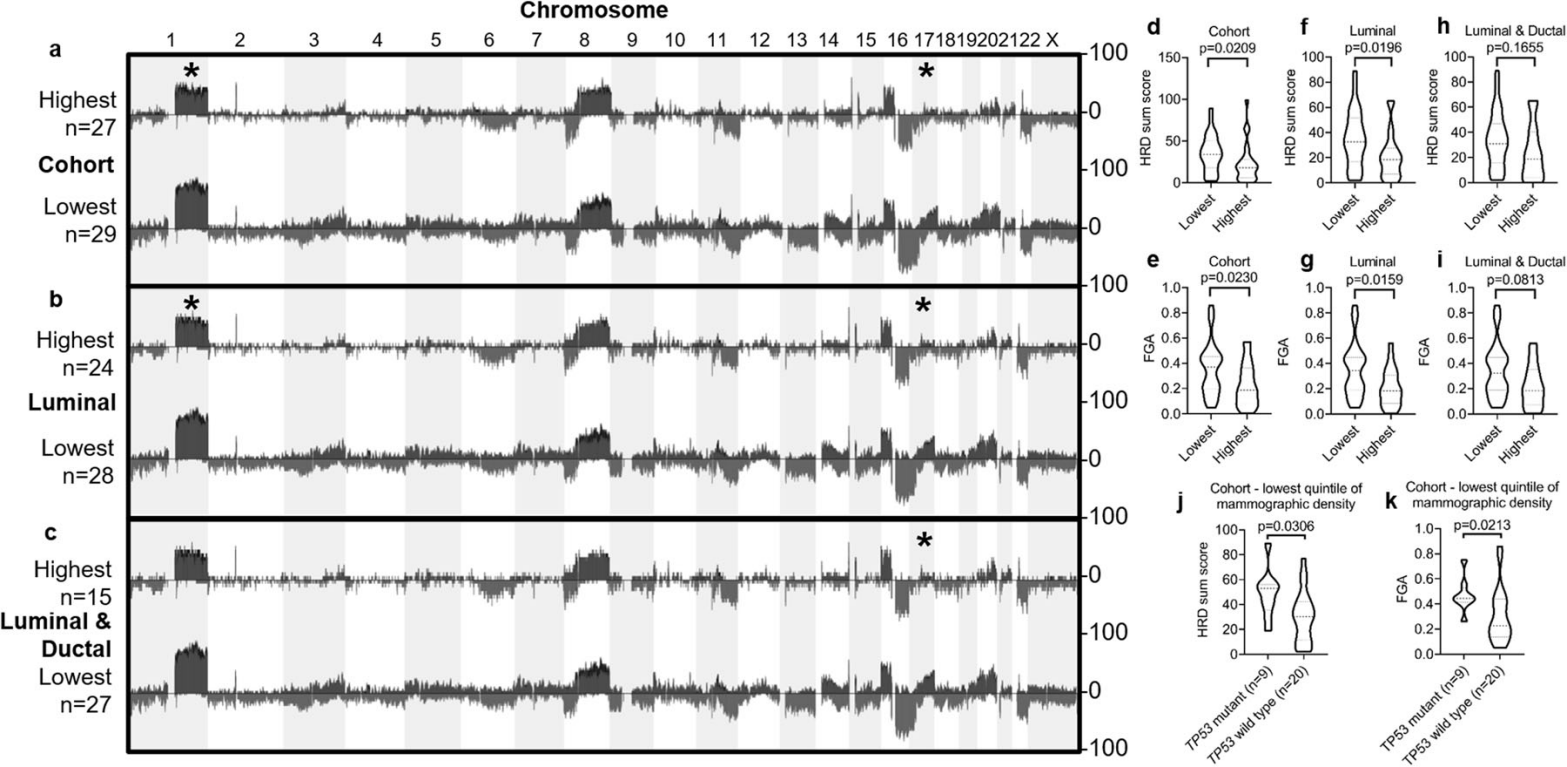
Copy number alterations in low and high mammographic dense breast cancers. Copy number aberrations are shown for 25 high and 30 low-MD breast cancers as the frequency of copy number changes in **a** the entire cohort, **b** luminal subtype, and **c** combined luminal/ductal subtype. The chromosome region highlighted with an asterisk represents a significant copy change between the two cohorts (thresholds of *p* < 0.05 and at least 25% frequency difference). Copy number profiles were used to generate a homologous recombination deficiency (HRD) sum score for **d** the entire cohort, **e** luminal subtype, and **f** combined luminal/ductal subtype. Copy number profiles were used to generate a fraction of the genome altered (FGA) score for **g** the entire cohort, **h** luminal subtype, and **i** combined luminal/ductal subtype. Both HRD (**j**) and FGA (**k**) were compared between *TP53* mutant and wild-type carcinomas in the lowest quintile of MD. Mann–Whitney test was applied to HRD and FGA violin plots.

Analysis of genome-wide copy number changes showed regions of copy number aberration that were significantly different in frequency between the lowest and highest quintiles of MD, with the specific loci differences shown in Supplementary Table 8. Copy number gains on chromosome 1q (1q32.2 and 1q41) and loss of 17p (17p12 and 17p13.2–13.3) were enriched in the lowest MD quintile cases compared to the highest MD quintile cases across the entire cohort (Fig. 2a) or if stratified by luminal subtype (Fig. 2b). Copy number loss on 17p was also significantly higher among the lowest MD quintile cancers when analysis was restricted to luminal cancers of ductal histology (Fig. 2c). Nine out of the ten cases with 17p LOH carried a *TP53* mutation.

The mean homologous recombination deficiency (HRD) sum score was significantly higher in the low-MD cancers within the entire cohort (mean score 35.2 versus 23.4, *p* = 0.0209, Fig. 2d) and luminal subtype (mean score 35.0 versus 21.1, *p* = 0.0196, Fig. 2e), but not combined luminal-ductal cancers (mean score 34.3 versus 24.2, *p* = 0.1655, Fig. 2f). The mean fraction of the genome altered (FGA) score was also significantly higher in the low-MD cancers within the entire cohort (mean score 0.36 versus 0.23, *p* = 0.0230, Fig. 2g) and luminal subtype (mean score 0.35 versus 0.21, *p* = 0.0159, Fig. 2h), but not combined luminal-ductal cancers (mean score 0.35 versus 0.22, *p* = 0.0813, Fig. 2i). *TP53* mutant cancers were compared to *TP53* wild-type cancers in the lowest quintile of MD and showed a significantly higher mean HRD sum score (mean score 49.0 versus 30.2, *p* = 0.0306, Fig. 2j) and FGA (mean score 0.47 versus 0.31, *p* = 0.0213, Fig. 2k).

To investigate the genomic basis of the difference in HRD and FGA between the low- and high-MD cancers, 28 genes involved in DNA repair (Supplementary Table 3) were sequenced in the breast cancers. Excluding *TP53*, the frequency of somatic mutations in individual genes nor the sum of somatic mutations in all DNA repair genes differed significantly between low-MD and high-MD cancers (Supplementary Table 9). Similarly, HRD high cancers (scores ≥ 42) showed no significant difference in the frequency of mutations in homologous recombination-linked genes compared to HRD low cancers (scores < 42) (Supplementary Table 9).

Of the 56 breast cancers sequenced, 12 (5 low MD and 7 high MD) had no detectable somatic driver gene mutations, but did harbor reliable HRD and FGA data indicating they were not poor samples due to low tumor purity.

### Differences in stromal tumor-infiltrating lymphocytes (TILs)

**Fig. 3:**
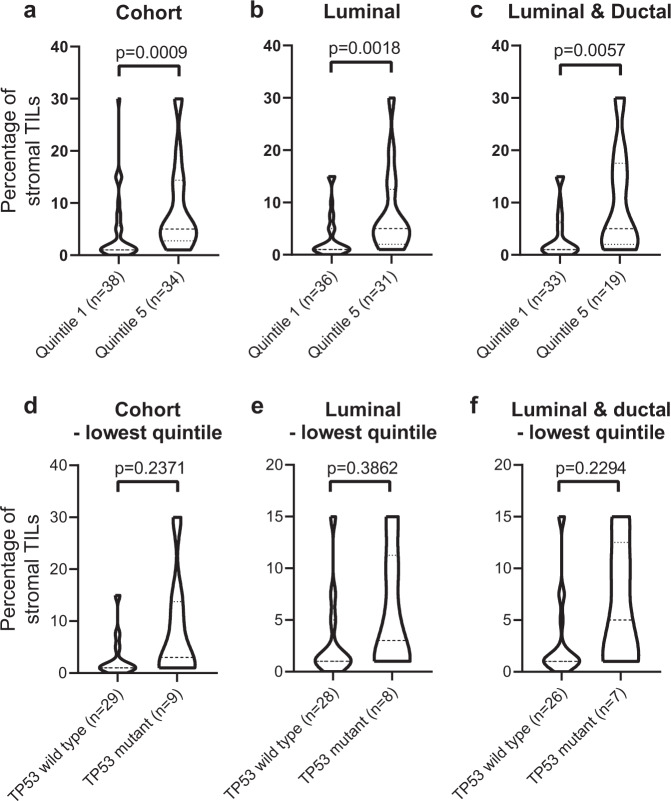
Comparing stromal tumor-infiltrating lymphocytes. Violin plots showing the percentage of stromal TILs in the tumor area for the full H&E-stained section. Percentage is scored comparing breast cancers in the lowest and highest quintiles of MD within **a** the entire breast cancer cohort, **b** luminal cancers only, **c** and combined luminal subtype and ductal histology cancers. Assessing breast cancers in the lowest quintile of MD only, the percentage of stromal TILS was scored comparing TP53 wild type and TP53 mutant breast cancers within **d** the entire breast cancer cohort, **e** luminal cancers only, **f** and combined luminal subtype and ductal histology cancers. Mann–Whitney test was applied.

Assessing an average percentage of stromal TILs/total area of intra-tumoral stromal area was performed and showed a significantly higher percentage of stromal TILs in the highest quintile compared to the lowest quintile of MD in the cohort as whole (Fig. 3a, *p* = 0.0009) or when stratified by luminal subtype (Fig. 3b, *p* = 0.0018) or luminal/ductal subtype (Fig. 3c, *p* = 0.0057). Additionally, the percentage of stromal TILs in *TP53* wild type and *TP53* mutant low-MD carcinomas were not significantly different in the cohort (Fig. 3d, *p* = 0.2371), luminal subtype (Fig. 3e, *p* = 0.3862), or luminal/ductal subtype (Fig. 3f, *p* = 0.2294); however, the overall number of *TP53* mutant cases is small and these data need to be interpreted cautiously.

## Discussion

Previous clinico-pathological analyses have shown that cancers arising in women with breasts of high versus low MD are very similar, but none have explored if this extends to the molecular genetic characteristics. In this study we report the largest and most detailed somatic genetic analysis of these two cohorts to gain insights into the mechanism of cancer predisposition associated with MD. The clinico-pathological features of our cohort are largely consistent with data reported in other studies^18,19^ showing cancers arising in the highest quintiles were more likely to be associated with younger age at diagnosis, an interval cancer diagnosis and a strong family history of breast cancer^20–22^.

The genomic data suggest that breast cancers arising in the highest and lowest quintiles of MD are very similar with respect to copy number profiles and overall frequency and spectrum of driver gene mutations but with the notable exception of *TP53*. The low frequency of *TP53* mutations observed in high-MD cancers may reflect the cancer growth promoting influence of the rich stromal microenvironment of dense breasts precluding the need for mutation of this strong somatic driver gene. One potential cancer-promoting factor in high-MD breast is tumor-promoting immune cells that have been shown in humans to be more prevalent in high- versus low-MD breasts^23^. Consistent with this we observed that among the entire cohort there was enrichment of TILs in the stroma of high-MD cancers compared to low-MD cancers. However, among the low-MD tumors where there were sufficient cases with *TP53* mutations, there was no difference in the percentage of stromal TILs between *TP53* wild type and mutant cases, suggesting that the immune microenvironment does not directly influence acquisition of *TP53* mutations.

The frequency of chromosome 17p loss was increased in the low-MD compared to high-MD cancers, even after adjustment for tumor histology. This pattern of genomic alteration is consistent with the *TP53* mutation data in low-MD cancers. *TP53* is located on 17p13.1 and is considered to be the major driver of 17p13.2-p13.3 loss in breast cancer, reflecting a reduced dosage of linked tumor suppressor genes within these regions^24,25^. The high HRD and FGA scores observed in low- compared to high-MD cancers appears largely driven by *TP53* mutant cases, consistent with previous observations from an analysis of pan-cancer TCGA data showing higher HRD and FGA in *TP53*-mutated tumors^26,27^.

There are still many gaps in the understanding of cellular and molecular mechanisms underlying the strong association of MD with breast cancer predisposition. While this study is the most comprehensive molecular analysis of low- and high-MD breast cancers, the overall numbers are small and these data need to be interpreted cautiously and will require validation in larger independent cohorts. Further insights might be gained by using additional image features of the dense tissue, and this may be the subject of future work.

## Methods

### Study cohort

Subjects were recruited from the Lifepool study (www.lifepool.org) which is a prospective cohort of women participating in population-based mammographic screening. The women were cancer-free at the time of recruitment. Epidemiological and mammographic screening data were available for all study subjects as reported previously^28^. Cancer incidence was determined by linkage to the Victorian Cancer Registry and BreastScreen Victoria. Pathology assessment such as hormone receptor status, Ki67, and tumor grade was extracted from diagnostic pathology reports where available. A strong family history of breast cancer was defined according to the Australian referral guidelines for breast cancer risk assessment (www.eviq.org.au) as either three first- or second-degree relatives with breast cancer; two first- or second-degree relatives with breast cancer (one diagnosed <50 years); male breast cancer any age; or ovarian cancer any age. All participants in this study gave written informed consent and research was approved by the Peter MacCallum Cancer Centre Human Ethics Committee under protocol #0966. All data are available as described in the Data Availability statement^29^.

### Automated measurement of breast density

Breast density measurements were attained from digital mammograms using AutoDensity^30,31^, which identifies the breast area in the digital mammogram (breast segmentation) and then classifies breast density by identifying distinctly white areas to be classified as “dense” (breast density segmentation). AutoDensity has been validated against Cumulus measurements, showing similar performances between the methods in terms of cancer risk, risk of interval breast cancers, and identifying the extreme quintiles of MD^32^. For this study, percent MD scores were generated from the most recent digital mammogram prior to breast tumor diagnosis. Scores were generated from the contralateral breast to cancer diagnosis and scores were adjusted for age at diagnosis.

### Tumor DNA extraction

For formalin-fixed paraffin-embedded (FFPE) tumors, a representative haematoxylin and eosin (H&E)-stained section was prepared and used as a template to needle-point micro-dissect cancer cells from subsequent 10-µm-thick-stained sections. DNA was extracted using the QIAamp DNA FFPE Tissue Kit (Qiagen, Valencia, CA, USA) and quantified using the Qubit dsDNA high-sensitivity assay kit (Thermo Fisher Scientific, Scoresby, Victoria Australia).

### Targeted sequencing and mutation detection

Targeted sequencing of tumor DNA was performed using a SureSelect XT Custom Panel (Agilent Technologies, Santa Clara, CA), targeting all exons and intron–exon boundaries of 13 hereditary breast and ovarian cancer genes, 28 DNA repair genes, and 27 genes commonly somatically mutated in breast cancer^28,33^ (Supplementary Table 1). Library preparation was performed from an input of 300 ng of tumor DNA using the KAPA Hyper Prep Kit (Kapa Biosystems, Wilmington, MA, USA). Sequencing of target-enriched DNA libraries was performed using the Illumina NextSeq500 generating paired-end 75 bp sequence reads. Somatic mutations in the tumor-sequencing data were identified by removing previously available germline variant data for HBOC panel genes, and where this information was not available, by applying the following filters: Transcript 1; quality ≥100; read depth ≥20; variants identified by at least two variant callers; minimum variant allele proportion ≥0.2; and minor allele frequency present at ≤0.0001 in the ExAc and GnomAD genomes database. Germline validation of genetic variants observed in tumors was performed on DNA extracted from either participant peripheral blood or saliva where available. Libraries were prepared using a custom-designed HaloPlex Targeted Enrichment Assay (Agilent Technologies, Santa Clara, CA)^34–36^. The variant data were filtered for loss-of-function mutations (defined as nonsense or frameshift or essential splice site mutations) and missense mutations that were classified as known pathogenic in ClinVar. Raw sequencing data are deposited in the NCBI Sequence Read Archive^37^.

### Genome-wide copy number analysis

Off-target sequencing reads from the tumor-sequencing data were used to generate genome-wide copy number data for 29 lowest quintile and 25 highest quintile breast cancers using CopywriteR^38^, utilizing a normal lymphocyte DNA control run in the same sequencing batch for the normalization baseline (NA12878, Coriell Institute). Data were imported into NEXUS Copy Number^TM^ (software v8.0 with build version 9169, BioDiscovery Inc.), segmented using a FASST2 segmentation algorithm, and visualized. Comparisons between groups were made using Nexus applying thresholds of *p* < 0.05 and at least 25% frequency difference. Within the Nexus software package gains were defined as log_2_ ratio >0.3 and losses at <−0.3. High copy number gains were called if the log_2_ ratio was >1.2 and homozygous deletions <−1.2.

### HRD and FGA scoring

Using the genome-wide copy number data, an HRD sum score^39^ was calculated for each tumor by summing the individual scores for telomeric allelic imbalances, large-scale state transitions, and loss-of-heterozygosity across all chromosomes^40,41^. The FGA was the number of bases affected by copy number change for each chromosome divided by the total base pair size of that chromosome and then averaged across all chromosomes^42^.

### p53 IHC and scoring

TMAs were constructed for Lifepool breast tumors with two independent 0.6 mm cores from each FFPE tumor tissue block. Sections were cut (4-µm-thick) and stained for p53 (clone DO-7; Leica Biosystems, Buffalo Grove, IL) using the Ventana Benchmark ULTRA automated slide processing system (Roche Diagnostics, Mannheim, Germany). Expression of p53 was scored across 170 breast cancers as overexpression (OE), complete absence (CA), cytoplasmic (CY), or wild type (WT) as described in Koebel el al.^43^. Normal or WT staining was characterized by variable staining intensity. Abnormal OE staining was strongly intense staining in >50% tumor cell nuclei. Abnormal CA staining was the complete absence of expression within tumor cell nuclei, with a variable intensity of normal p53 expression seen in fibroblasts and lymphocytes acting as an intrinsic control. Abnormal CY staining was diffuse cytoplasmic staining in the absence of strong nuclear staining.

### TIL evaluation

Stromal TILs were assessed for all cases in the highest and lowest quintile of MD using one H&E-stained section of the whole FFPE block. The evaluation was conducted according to the standardized approach for TIL evaluation in breast cancer from the TILs working group^44^. In short, mononuclear stromal TILs within the borders of the invasive tumor were evaluated but excluding areas of necrosis, previous core biopsy sites, and granulocytes. The estimation was semi-quantitative, assessing an average percentage of stromal TILs in the tumor area for the full section, with no evaluation of hotspots. Independent assessment was performed by D.C. and D.B., and a consensus was reached for discordant cases.

### Statistical analysis

A two-tailed *t*-test (continuous data), two-tailed Fisher’s exact test (2 × 2 and 3 × 2 tables), or chi-square test (4 × 2 or greater tables) were applied to compare clinico-pathological differences between the highest and lowest quintiles of MD. MD quintiles were selected prior to the analysis conducted for this study, as done by others^45–47^. Mann–Whitney tests were used to calculate differences in FGA, HRD, and mutation burden between the two density cohorts. Error bars represent the standard deviation.

### Reporting summary

Further information on research design is available in the Nature Research Reporting Summary linked to this article.

## Supplementary information

Supplementary information

Reporting Summary

Supplementary Dataset

## Data Availability

The DNA sequencing data generated during the study are publicly available in the NCBI Sequence Read Archive (SRA): https://identifiers.org/ncbi/insdc.sra:SRP269052^37^. All other datasets generated and analyzed during the current study will be made available on reasonable request, from the corresponding author D.C., as described in the following figshare metadata record: 10.6084/m9.figshare.12601754 (ref. ^29^).
