# Supplementary material for: The TP53 mutation rate differs in breast cancers that arise in women with high or low mammographic density
Source: NPJ Breast Cancer. 2020 Aug 7;6:34. doi: 10.1038/s41523-020-00176-7 (PMC7414106; doi:10.1038/s41523-020-00176-7)
Supplement: Supplementary file 1 — Supplementary information [file 41523_2020_176_MOESM1_ESM.pdf]

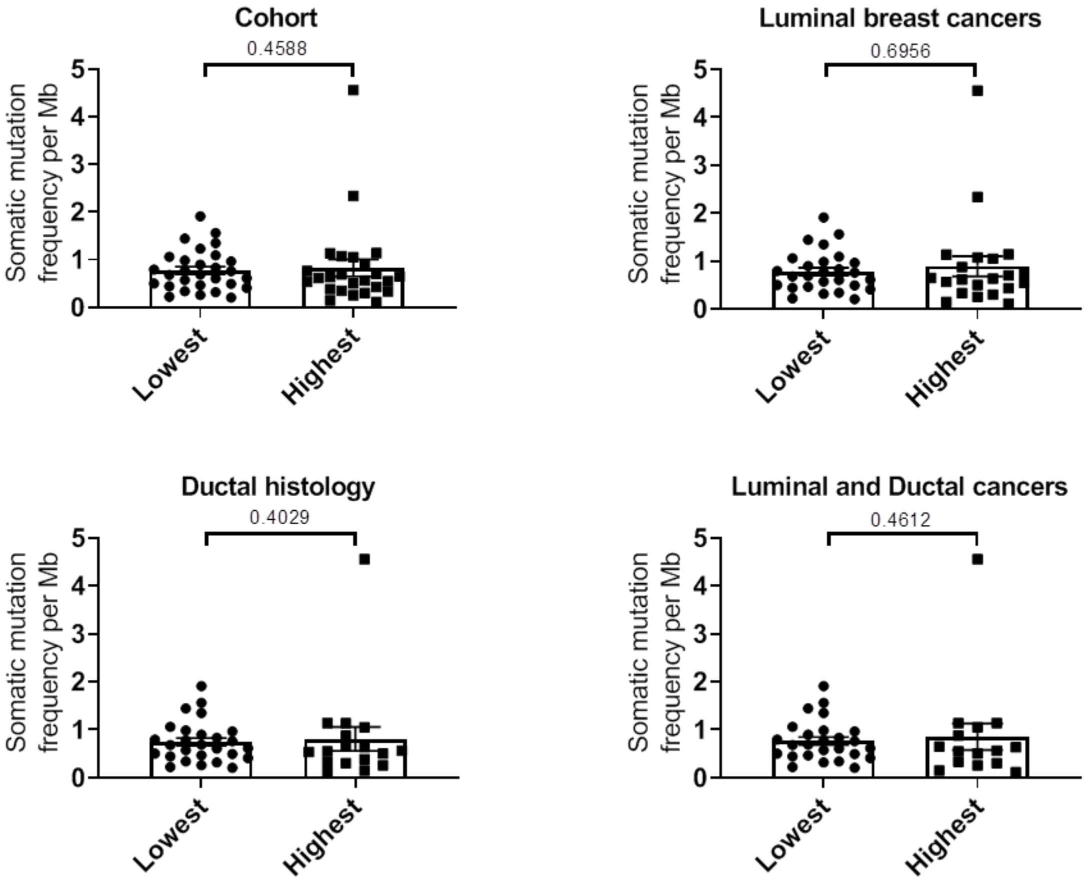

Supplementary figure 1. Mutation burden of cancers arising in the lowest and highest quintiles of mammographic density. The somatic mutation frequency (frameshift variants, stop gains/loss, splice variants, missense variants, inframe deletion/insertion and synonymous variants) per Mb was calculated comparing the lowest and highest quintiles for (**A**) the entire cohort, (**B)** luminal breast cancer, (**C**) Ductal breast cancers and (**D)** cancers that are both luminal and ductal. Mann–Whitney tests were applied, no significant differences were observed.

Supplementary table 1. Breast cancer subtypes diagnosed in the Lifepool cohort. Two tailed t-test was applied comparing lowest and highest MD breast cancers

Tumour subtype	Lowest (n=142)	Highest (n=119)	*P* value
Invasive lobular carcinom	7 (5%)	11 (9%)	>0.9999
Invasive ductal carcinoma	117 (82%)	91 (76%)	>0.9999
Mucinous carcinoma	2 (1%)	3 (3%)	>0.9999
Mixed carcinoma	5 (4%)	5 (4%)	>0.9999
Information not available	5 (4%)	4 (3%)	>0.9999
Invasive tubular carcinom	2 (1%)	2 (2%)	>0.9999
Papillary carcinoma	1 (1%)	1 (1%)	>0.9999
Metaplastic carcinoma	1 (1%)	1 (1%)	>0.9999
Adenocarcinoma	1 (1%)	1 (1%)	>0.9999
Granular cell tumour	1 (1%)	0	>0.9999

Supplementary table 2. Clinico-pathological features of in situ breast tumours diagnosed in the lowest and highest quintiles of mammographic density. Calculation of percentage within the cohort is shown within parentheses. Strong family history of breast cancer was defined as either three first or second degree relatives with breast cancer; two first or second degree relatives with breast cancer (one diagnosed <50 years); male breast cancer any age; or ovarian cancer any age. NA, data not available. a two-tailed t-test was applied; b two-tailed Fisher's exact test was applied.

**Characteristics**	**Lowest**	**Highest**	***P* value**
In Situ	31	58	
**Age at diagnosis**			
Mean ± SD	63.2 ± 7.8	59.5 ± 7.8	0.0562a
Median	63.4	58.8	
Range	48 - 81	43 - 75	
**Tumour size**			
Mean ± SD	26.6 ± 25.3	21.5 ± 20.2	0.5076a
Median	20	12	
Range	2.5 - 90	1.5 - 80	
**Estrogen receptor status**			
Negative	2 (22%)	7 (24%)	>0.9999b
Positive	7 (78%)	22 (76%)	
NA	22	29	
**Tumoir histology subtype**			
DCIS	25 (81%)	47 (81%)	>0.9999b
Other	6 (19%)	11 (19%)	
**Tumour grade**			
Low	2 (10%)	7 (19%)	0.5845b
Intermediate	6 (29%)	7 (19%)	
High	13 (61%)	23 (62%)	
NA	10	21	
**First degree relatives with breast cancer**			
Yes	9 (29%)	11 (19%)	0.2975b
No	22 (71%)	47 (81%)	
**Strong family history of breast cancer**			
Yes	5 (16%)	7 (12%)	0.7459b
No	26 (84%)	51 (88%)	

Supplementary table 3. Genes analysed on the breast cancer sequencing panel. The 27 somatic driver genes, 13 HBOC genes and 28 DNA repair genes were included on the Agilent Sureselect sequencing panel.

***HBOC* gene**	**Somatic driver**	**DNA repair genes**	**Associated repair pathway**
ATM	*AKT1*	*ATM*	NER
BRCA1	*ARID1A*	*BAP1*	Indirect
BRCA2	*BAP1*	*BLM*	Indirect
BRIP1	*BRCA1*	*BRCA1*	NER
CDH1	*BRCA2*	*BRCA2*	HR/FA
CHEK2	*CBFB*	*BRIP1*	FA
NTHL1	*CDH1*	*CHEK2*	NER
PALB2	*CDKN2A*	*DDB2*	NER
PTEN	*CHEK2*	*ERCC2*	NER
RAD51C	*ERBB2*	*ERCC3*	NER
RAD51D	*FOXP1*	*ERCC4*	NER
STK11	*GATA3*	*ERCC5*	NER
TP53	*KMT2C*	*FANCA*	FA
	KRAS	*FANCC*	FA
	MAP2K4	*FANCG*	FA
	MAP3K1	*MLH1*	MMR
	MEN1	*MSH2*	MMR
	NCOR1	*MSH6*	MMR
	NF1	*MUTYH*	BER
	PIK3CA	*NBN*	NER
	PIK3R1	*PALB2*	HR/FA
	PTEN	*PMS2*	MMR
	RB1	*RAD51C*	HR/FA
	RUNX1	*RAD51D*	NER
	SF3B1	*RECQL4*	Indirect
	TBX3	*TP53*	Indirect
	TP53	*XPA*	NER
	ATM	*XPC*	NER
	BRCA1		
	BRCA2		
	BRIP1		
	CDH1		
	CHEK2		
	PALB2		
	PTEN		
	RAD51C		
	RAD51D		
	STK11		
	TP53		

Supplementary table 4. Breast tumours sequenced on the Agilent SureSelect Panel compared for age and grade. A two-tailed t-test was applied; b two-tailed Fisher's exact test was applied.

Characteristics	Lowest	Highest	*P* value
**Age at diagnosis**			
Mean ± SD	63.2 ± 8.2	60.7 ± 8.0	0.2570a
Median	65.8	61.5	
Range	42-79	43-75	
**Tumour grade**			
1	6	6	0.5925b
2	14	16	
3	9	5	

Supplementary table 5. Breast cancer somatic driver mutation profile in triple negative and lobular breast cancers diagnosed in the highest and lowest quintiles of MD.A two-tailed p-value was calculated

		Triple negative breast cancers (Lowest n=1, Highest n=3)							Lobular breast cancers (Lowest n=1, Highest n=4)						
		Lowest n (%)			Highest n (%)				Lowest n (%)			Highest n (%)			
**Gene**	**Total**	**Lof**	**MS**	**%**	**Lof**	**MS**	**%**	**P**	**Lof**	**MS**	**%**	**Lof**	**MS**	**%**	**P**
CDH1	5	-	-	-	-	-	-	-	1	-	100%	4	-	100%	>0.9999
PIK3CA	4	-	-		-	-		-	-	1	100%	-	3	75%	>0.9999
TP53	2	-	-	-	1	-	33%	>0.9999	-	1	100%	-	-	-	0.2
CBFB	2	-	-	-	-	-	-	-	-	1	100%	1	-	25%	0.4
ARID1A	1	-	-	-	-	-	-	-	-	-	-	-	1	25%	>0.9999
NCOR1	1	-	-	-	-	-	-	-	-	-	-	1	-	25%	>0.9999
BRCA2	1	-	-	-	-	-	-	-	1	-	100%	-	-	-	0.2

Supplementary table 6. Clinico-pathological features of TP53 wiltype and mutant breast carcinomas diagnosed in the lowest quintile of mammographic density.

Characteristics	TP53 mutant	TP53 wild type	*P value*
Invasive	9	133	
**Age at diagnosis**			
Mean ± SD	64.6 ± 6.9	64.2 ± 6.8	0.9623a
Median	66.9	65.6	
Range	55 - 74	50 - 88	
**Screening timing**			
Lapsed screener	0	2 (2%)	0.2339b
Interval cancer	2 (22%)	8 (6%)	
Screen detected	7 (78%)	123 (92%)	
**Tumour size**			
<20mm	3 (33%)	77 (63%)	0.0354b
20 - 49mm	3 (33%)	36 (29%)	
≥50mm	3 (33%)	10 (8%)	
NA	0	10	
**Intrinsic subtype**			
TNBC	1 (11%)	9 (8%)	0.8198c
ER−, Her2+	0	2 (2%)	
Luminal Her2+	0	8 (7%)	
Luminal	8 (89%)	96 (83%)	
NA	0	8	
**Invasive cancer histology subtype**			
Ductal	8 (89%)	99 (74%)	0.3407b
Lobular	1 (11%)	6 (5%)	
Other invasive	0	18 (14%)	
**Tumour grade**			
G1	0	34 (28%)	0.0028b
G2	2 (22%)	60 (49%)	
G3	7 (78%)	29 (24%)	
NA	0	10	
**Nodal status**			
Positive	3 (43%)	19 (20%)	0.1617b
Negative	4 (57%)	78 (80%)	
NA	2	36	
**First degree relatives with breast cancer**			
Yes	4 (44%)	35 (26%)	0.2587b
No	5 (56%)	98 (74%)	
**Strong family history of breast cancer**			
Yes	2 (22%)	9 (7%)	0.1456b
No	7 (78%)	124 (93%)	

Supplementary table 7. Somatic driver and DNA repair gene muation profile in TP53 mutant and wild type cancers in the lowest quintile of mammographic density. Genes highlighted in grey represent DNA repair genes. A two-tailed p-value was calculated.

	Entire cohort (Mutant n=9, Wild type n=20)						
	Lowest n (%)			Highest n (%)			
**Gene**	**Lof**	**MS**	**%**	**Lof**	**MS**	**%**	***P***
AKT1	-	1	11%	-	1	5%	>0.9999
ARID1A	1	-	11%	1	1	10%	>0.9999
CBFB	1	1	22%	3	-	15%	>0.9999
CDH1	1	-	11%	1	1	10%	>0.9999
GATA3	-	-		2	-	10%	0.5567
KMT2C	-	-		3	1	20%	0.2800
MAP2K4	-	-		1	-	5%	>0.9999
MAP3K1	-	-		6	1	35%	0.0661
NCOR1	-	1	11%	-	-	-	0.3103
PIK3CA	-	5	56%	-	11	55%	>0.9999
PTEN	-	-		1	1	10%	0.5567
RUNX1	-	-		-	1	5%	>0.9999
SF3B1	-	-		-	1	5%	>0.9999
STK11	-	1	11%	-	-	-	0.3103
TBX3	1		11%	2	-	10%	>0.9999
ATM	-	-		1	1	10%	0.5567
BAP1	-	-		-	1	5	>0.9999
BRCA2	-	-		-	2	10%	0.5567
CHEK2	-	-		-	1	5%	>0.9999
FANCA	-	-		-	1	5%	>0.9999
FANCC	-	-		1	1	10%	0.5567
PMS2	-	-		-	3	15%	0.5320
XPA	-	1	11%	-	-	-	0.3103

Supplementary table 8. Comparative P-values for comrosomsome 1q and 17p differences.

Region	Cytoband Location	CN Event	Region Length	lowest quintile (%)	highest quintile (%)	Difference %	p-value	Genes affected within region
**Entire cohort**								
chr17:0-1,100,000	p13.3	CN Loss	1100000	57	12	45	7.20E-04	DOC2B, LOC100506371, RPH3AL, LOC100506388, C17orf97, FAM101B, VPS53, FAM57A, GEMIN4, DBIL5P, GLOD4, RNMTL1, NXN, TIMM22, MIR3183, ABR
chr17:15,350,000-15,400,000	p12	CN Loss	50000	63	16	47	7.93E-04	CDRT4, TVP23C-CDRT4
chr17:15,625,000-15,979,078	p12	CN Loss	354078	67	20	47	9.34E-04	TBC1D26, CDRT15P2, MEIS3P1, LOC101928567, ADORA2B, ZSWIM7, TTC19, NCOR1
chr1:210,001,317-210,225,278	q32.2	CN Gain	223961	90	44	46	3.42E-04	DIEXF, SYT14
chr1:210,225,278-210,573,280	q32.2	CN Gain	348002	90	48	42	9.13E-04	SYT14, SERTAD4-AS1, SERTAD4, HHAT
chr1:214,513,946-215,000,000	q41	CN Gain	486054	90	44	46	3.42E-04	PTPN14, CENPF
chr1:215,000,000-215,206,748	q41	CN Gain	206748	90	48	42	9.13E-04	KCNK2
**Luminal type**								
chr17:0-1,100,000	p13.3	CN Loss	1100000	61	10	51	3.17E-04	DOC2B, LOC100506371, RPH3AL, LOC100506388, C17orf97, FAM101B, VPS53, FAM57A, GEMIN4, DBIL5P, GLOD4, RNMTL1, NXN, TIMM22, MIR3183, ABR
chr17:13,404,453-13,750,000	p12	CN Loss	345547	64	14	50	5.40E-04	HS3ST3A1
chr17:15,150,000-15,350,000	p12	CN Loss	200000	64	14	50	5.40E-04	PMP22, MIR4731, TEKT3, CDRT4, TVP23C-CDRT4
chr17:2,350,000-2,450,000	p13.3	CN Loss	100000	64	14	50	5.40E-04	METTL16
chr17:3,950,000-4,000,000	p13.2	CN Loss	50000	64	14	50	5.40E-04	ZZEF1
chr17:5,494,300-5,500,000	p13.2	CN Loss	5700	64	14	50	5.40E-04	
chr17:12,350,000-12,700,000	p12	CN Loss	350000	68	14	54	3.65E-04	LINC00670, LOC101928418, MYOCD, LOC100128006, ARHGAP44
chr17:13,100,000-13,404,453	p12	CN Loss	304453	68	14	54	3.65E-04	HS3ST3A1
chr17:15,350,000-15,400,000	p12	CN Loss	50000	68	14	54	3.65E-04	CDRT4, TVP23C-CDRT4
chr1:210,001,317-210,573,280	q32.2	CN Gain	571963	93	48	45	6.82E-04	DIEXF, SYT14, SERTAD4-AS1, SERTAD4, HHAT
chr1:214,513,946-215,000,000	q41	CN Gain	486054	93	48	45	6.82E-04	PTPN14, CENPF
**Ductal and luminal type**								
chr17:0-1,100,000	p13.3	CN Loss	1100000	59	7	53	9.53E-04	DOC2B, LOC100506371, RPH3AL, LOC100506388, C17orf97, FAM101B, VPS53, FAM57A, GEMIN4, DBIL5P, GLOD4, RNMTL1, NXN, TIMM22, MIR3183, ABR
chr17:13,750,000-14,250,000	p12	CN Loss	500000	59	7	53	9.53E-04	CDRT15P1, COX10-AS1, COX10, CDRT15, MGC12916, HS3ST3B1
chr17:13,404,453-13,750,000	p12	CN Loss	345547	63	7	56	3.96E-04	HS3ST3A1
chr17:15,150,000-15,350,000	p12	CN Loss	200000	63	7	56	3.96E-04	PMP22, MIR4731, TEKT3, CDRT4, TVP23C-CDRT4
chr17:13,100,000-13,404,453	p12	CN Loss	304453	67	7	60	2.35E-04	HS3ST3A1
chr17:15,350,000-15,400,000	p12	CN Loss	50000	67	7	60	2.35E-04	CDRT4, TVP23C-CDRT4

Supplementary table 9. DNA repair pathway mutation profile. A two-tailed p-value was calculated. Bold black boxes highlight DNA repair genes muations that were significantly different between low and high MD breast cancers or low and high HRD cancers.

		Entire cohort (Lowest n=29, Highest n=27)							Luminal breast cancers (Lowest n=28, Highest n=24)							Luminal & ductal breast cancers (Lowest n=27, Highest n=15)							HRD sum score (Below 42 n=41, Above 42 n=15)						
		Lowest n (%)			Highest n (%)				Lowest n (%)			Highest n (%)				Lowest n (%)			Highest n (%)				Lowest n (%)			Highest n (%)			
**DNA repair pathway**	**Gene**	**Lof**	**MS**	**%**	**Lof**	**MS**	**%**	***P***	**Lof**	**MS**	**%**	**Lof**	**MS**	**%**	***P***	**Lof**	**MS**	**%**	**Lof**	**MS**	**%**	***P***	**Lof**	**MS**	**%**	**Lof**	**MS**	**%**	***P***
**Nucleotide excision repair**	**All**	-	1	3%	-	1	4%	>0.9999	-	1	4%	-	1	4%	>0.9999	-	1	4%	-	-	-	>0.9999	-	1	2%	-	1	7%	>0.9999
	ERCC4	-	-	-	-	1	4%	0.4821	-	-	-	-	1	4%	0.4615	-	-	-	-	-	-	-	-	-	-	-	1	7%	0.2679
	XPA	-	1	3%	-	-	-	>0.9999	-	1	4%	-	-	-	>0.9999	-	1	4%	-	-	-	>0.9999	-	1	2%	-	-	-	>0.9999
**Homologous recombination**	**All**	1	3	14%	-	3	11%	>0.9999	1	3	14%	-	3	13%	>0.9999	1	3	15%	-	2	13%	>0.9999	-	6	15%	1	1	13%	>0.9999
	ATM	1	1	7%	-	1	4%	>0.9999	1	1	7%	-	1	4%	>0.9999	1	1	7%	-	-	-	0.5296	-	2	5%	1	-	7%	>0.9999
	BRCA1	-	-	-	-	2	7%	0.2279	-	-	-	-	2	8%	0.2081	-	-	-	-	2	13%	0.1220	-	1	2%	-	1	7%	>0.9999
	BRCA2	-	1	3%	-	-	-	>0.9999	-	1	4%	-	-	-	>0.9999	-	1	4%	-	-	-	>0.9999	-	1	2%	-	-	-	>0.9999
	CHEK2	-	1	3%	-	-	-	>0.9999	-	1	4%	-	-	-	>0.9999	-	1	4%	-	-	-	>0.9999	-	1	2%	-	-	-	>0.9999
**Fanconi anaemia pathway**	**All**	2	1	10%	1	1	7%	>0.9999	1	-	4%	1	1	8%	0.5895	1	1	7%	1	1	13%	0.6080	1	2	7%	1	1	13%	0.6023
	BRIP1	-	-	-	-	1	4%	0.4821	-	-	-	-	1	4%	0.4615	-	-	-	-	1	7%	0.3571	-	1	2%	-	-	-	>0.9999
	FANCA	1	-	3%	1	-	4%	>0.9999	1	-	4%	1	-	4%	>0.9999	1	-	4%	1	-	7%	>0.9999	-	1	2%	-	-	-	>0.9999
	FANCC	1	1	7%	-	-	-	0.4916	-	-	-	-	-	-	-	-	-	-	-	-	-	-	1	-	2%	1	1	13%	0.1717
**Indirect**	**All**	3	6	31%	2	1	11%	0.1043	2	6	29%	1	-	4%	**0.0281**	2	5	26%	1	-	7%	0.2225	3	3	15%	2	4	40%	0.0644
	BAP1	-	1	3%	-	-	0%	>0.9999	-	1	4%	-	-	-	>0.9999	-	1	4%	-	-	-	>0.9999	-	1	2%	-	-	-	>0.9999
	BLM	-	-	-	-	1	4%	0.4821	-	-	-	-	1	4%	0.4615	-	-	-	-	-	-	-	-	1	2%	-	-	-	>0.9999
	RECQL4	-	-	-	1	-	4%	0.4821	-	-	-	1	-	4%	0.4615	-	-	-	1	-	7%	0.3571	1	-	2%	-	-	-	>0.9999
	TP53	3	6	31%	1	1	7%	**0.0420**	2	6	29%	-	-	-	**0.0051**	2	5	26%	-	-	-	0.0772	2	2	10%	2	5	47%	**0.0049**
**DNA repair sum**	**All**	6	11	59%	3	6	33%	0.0677	4	10	50%	2	5	29%	0.1621	4	10	52%	2	3	33%	0.3371	4	12	39%	4	7	73%	**0.0344**
