# Supplementary material for: The TP53 mutation rate differs in breast cancers that arise in women with high or low mammographic density
Source: NPJ Breast Cancer. 2020 Aug 7;6:34. doi: 10.1038/s41523-020-00176-7 (PMC7414106; doi:10.1038/s41523-020-00176-7)
Supplement: Supplementary file 2 — Reporting Summary [file 41523_2020_176_MOESM2_ESM.pdf]

nature research

Corresponding author(s): Dane Cheasley

Last updated by author(s): Jul 8, 2020

Reporting Summary

Nature Research wishes to improve the reproducibility of the work that we publish. This form provides structure for consistency and transparency in reporting. For further information on Nature Research policies, see our Editorial Policies and the Editorial Policy Checklist.

Statistics

For all statistical analyses, confirm that the following items are present in the figure legend, table legend, main text, or Methods section.

n/a	Confirmed	
☐	☒	The exact sample size (n) for each experimental group/condition, given as a discrete number and unit of measurement
☒	☐	A statement on whether measurements were taken from distinct samples or whether the same sample was measured repeatedly
☐	☒	The statistical test(s) used AND whether they are one- or two-sided<br>*Only common tests should be described solely by name; describe more complex techniques in the Methods section.*
☒	☐	A description of all covariates tested
☐	☒	A description of any assumptions or corrections, such as tests of normality and adjustment for multiple comparisons
☐	☒	A full description of the statistical parameters including central tendency (e.g. means) or other basic estimates (e.g. regression coefficient) AND variation (e.g. standard deviation) or associated estimates of uncertainty (e.g. confidence intervals)
☐	☒	For null hypothesis testing, the test statistic (e.g. F, t, r) with confidence intervals, effect sizes, degrees of freedom and P value noted<br>*Give P values as exact values whenever suitable.*
☒	☐	For Bayesian analysis, information on the choice of priors and Markov chain Monte Carlo settings
☒	☐	For hierarchical and complex designs, identification of the appropriate level for tests and full reporting of outcomes
☒	☐	Estimates of effect sizes (e.g. Cohen's d, Pearson's r), indicating how they were calculated

Our web collection on statistics for biologists contains articles on many of the points above.

Software and code

Policy information about availability of computer code

Data collection	No software was used
Data analysis	No software was used

For manuscripts utilizing custom algorithms or software that are central to the research but not yet described in published literature, software must be made available to editors and reviewers. We strongly encourage code deposition in a community repository (e.g. GitHub). See the Nature Research guidelines for submitting code & software for further information.

Data

Policy information about availability of data

All manuscripts must include a data availability statement. This statement should provide the following information, where applicable:

- Accession codes, unique identifiers, or web links for publicly available datasets
- A list of figures that have associated raw data
- A description of any restrictions on data availability

The DNA sequencing data generated during the study are publicly available in the NCBI Sequence Read Archive (SRA): https://identifiers.org/ncbi/insdc.sra:SRP269052. All other datasets generated and analysed during the current study, will be made available on reasonable request, from the corresponding author Dr. Dane Cheasley, as described in the following figshare metadata record: https://doi.org/10.6084/m9.figshare.12601754.

Field-specific reporting

Please select the one below that is the best fit for your research. If you are not sure, read the appropriate sections before making your selection.

☒ Life sciences ☐ Behavioural & social sciences ☐ Ecological, evolutionary & environmental sciences

For a reference copy of the document with all sections, see nature.com/documents/nr-reporting-summary-flat.pdf

Life sciences study design

All studies must disclose on these points even when the disclosure is negative.

Sample size	No sample size calculation was performed. We analyzed all available Lifepool breast cancers in the lowest and highest quintiles of mammographic density.
Data exclusions	Breast cancer samples with a germline mutation in a hereditary breast or ovarian cancer gene were excluded from the study.
Replication	All raw sequencing data has been uploaded to the Sequence Read Archive (SRA) to enable replication of data.
Randomization	Breast cancer samples that were in the highest on lowest quintiles of mammographic density were allocated to the study.
Blinding	Investigators were blinded to density measurements during genomic analysis of each samples to avoid bias.

Reporting for specific materials, systems and methods

We require information from authors about some types of materials, experimental systems and methods used in many studies. Here, indicate whether each material, system or method listed is relevant to your study. If you are not sure if a list item applies to your research, read the appropriate section before selecting a response.

Materials & experimental systems

n/a	Involved in the study	
☐	☒	Antibodies
☒	☐	Eukaryotic cell lines
☒	☐	Palaeontology and archaeology
☒	☐	Animals and other organisms
☐	☒	Human research participants
☒	☐	Clinical data
☒	☐	Dual use research of concern

Methods

n/a	Involved in the study	
☒	☐	ChIP-seq
☒	☐	Flow cytometry
☒	☐	MRI-based neuroimaging

Antibodies

Antibodies used	p53 (clone DO-7, Leica Biosystems, Buffalo Grove, IL)
Validation	Immunohistochemistry optimization and expression scoring of 170 breast cancers was performed as described and validated in Koebel el al 2016 (doi:10.1002/cjp2.53), which is referenced in the methods.

Human research participants

Policy information about studies involving human research participants

Population characteristics	Women were breast cancer-free at time of recruitment. Epidemiological and mammographic screening data were available for all study subjects by linkage to the Victorian Cancer Registry and BreastScreen Victoria. Pathology assessment such as hormone receptor status, Ki67 and tumor grade was extracted from diagnostic pathology reports where available. Family history of cancer was recorded by Lifepool via a questionnaire sent to participants.
Recruitment	Subjects were recruited from the Lifepool study (www.lifepool.org) which is a prospective cohort of women participating in population-based mammographic screening in Australia
Ethics oversight	All participants in this study gave written informed consent and research was approved by the Peter MacCallum Cancer Centre Human Ethics Committee under ethics approval #0966.

Note that full information on the approval of the study protocol must also be provided in the manuscript.
